# Supplementary material for: A Double Mutation in the ALS Gene Confers a High Level of Resistance to Mesosulfuron-Methyl in Shepherd’s-Purse
Source: Plants (Basel). 2023 Jul 23;12(14):2730. doi: 10.3390/plants12142730 (PMC10386087; doi:10.3390/plants12142730)
Supplement: Supplementary file 1 [file plants-12-02730-s001.zip › plants-2469408-supplementary.pdf]

Supplementary

A Double Mutation in the *ALS* Gene Confers a High Level of Resistance to Mesosulfuron-Methyl in Shepherd's-Purse

Huan Lu, Yingze Liu, Dexiao Bu, Fan Yang, Zheng Zhang, Sheng Qiang*

Weed Research Laboratory, College of Life Sciences, Nanjing Agricultural University, Nanjing 210095, China

*Correspondence to: wrl@njau.edu.cn

Table S1. Interaction energy between mesosulfuron-methyl and different ALS protein isoforms in the S (WT) and R (mutant) plants.

	Interaction energy (kcal/mol)
WT-isoform I	-58.66
Mutant-isoform I	-42.79
WT-isoform II	-56.18
Mutant-isoform II	-47.39

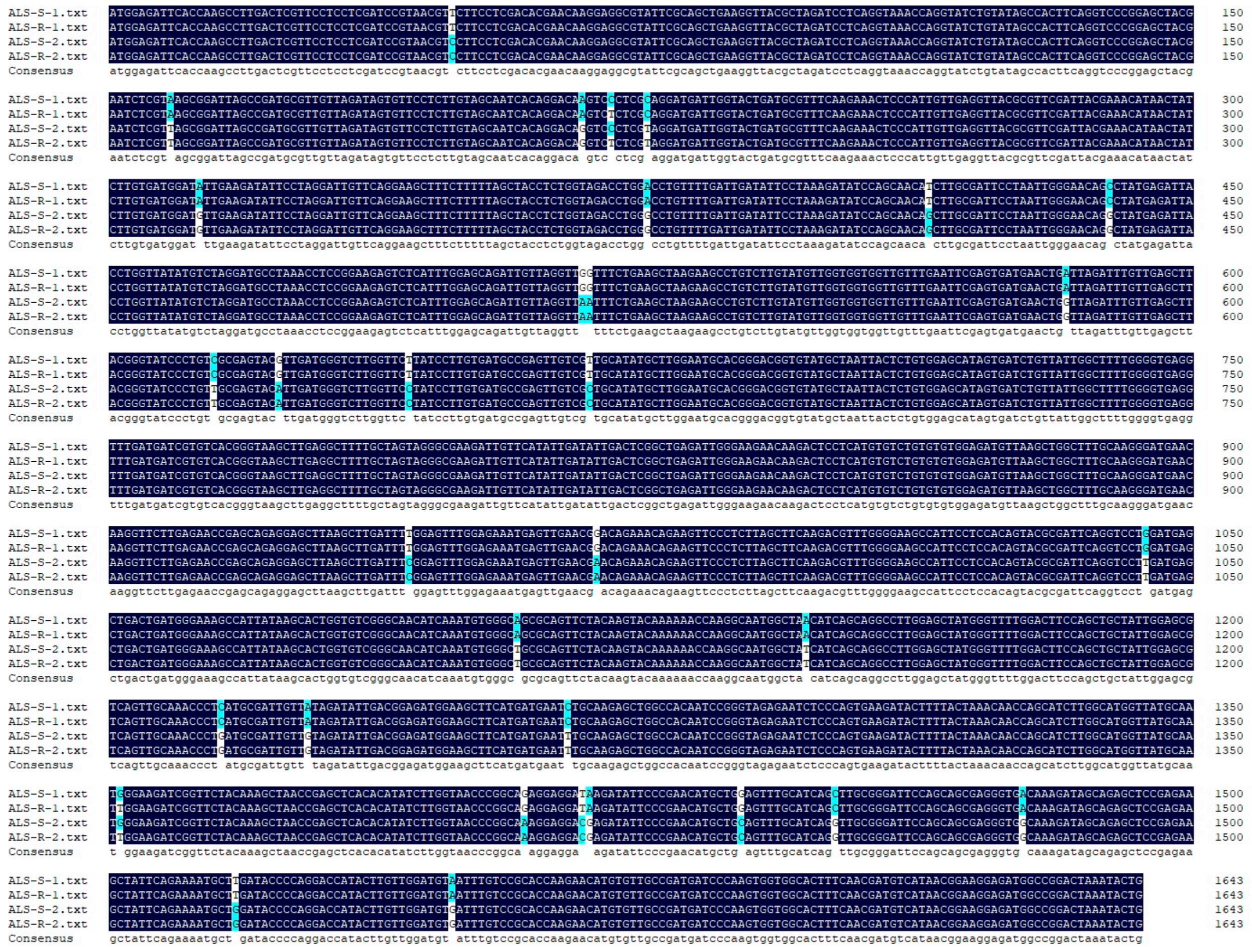

Figure S1. Sequence comparisons of two *ALS* gene copies in the S and R Shepherd's-Purse plants (ALS-S-1 and ALS-S-2 represent two *ALS* gene copies in the S plants, ALS-R-1 and ALS-R-2 represent two *ALS* gene copies in the R plants)

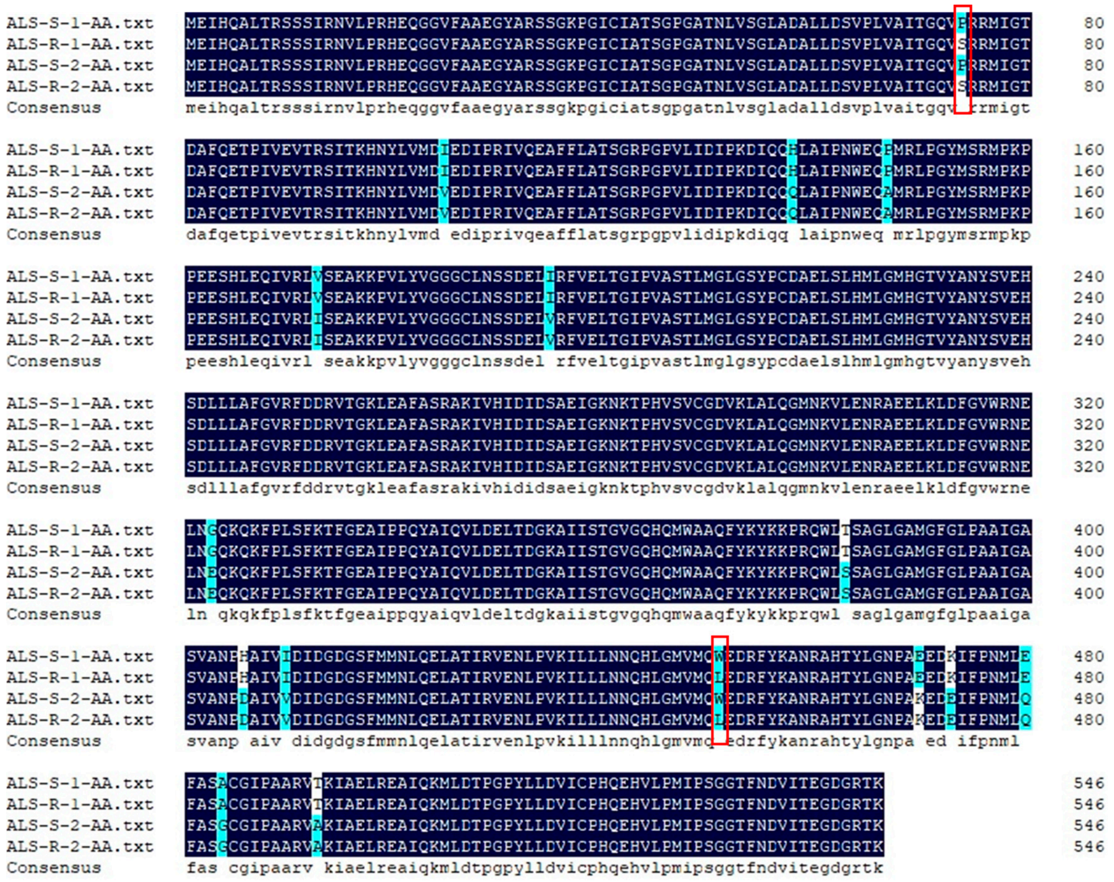

Figure S2. Alignment of two ALS protein isoforms in the S and R Shepherd's-Purse plants (ALS-S-1-AA and ALS-S-2-AA represent two ALS protein isoforms in the S plants, ALS-R-1-AA and ALS-R-2-AA represent two ALS protein isoforms in the R plants, two red boxes showed Pro197-Ser mutation and Trp574-Leu mutation, respectively)

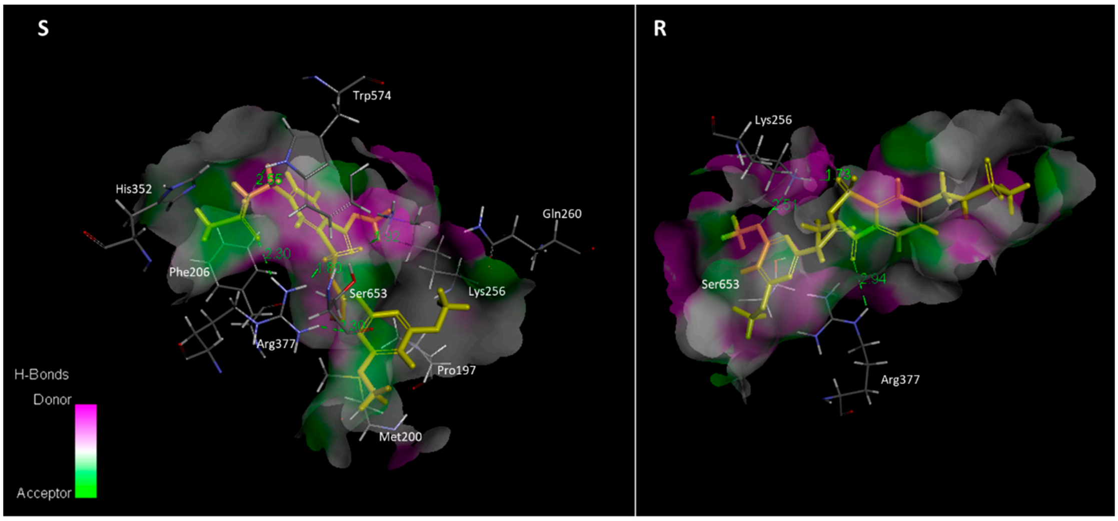

Figure S3. Spatial structure of contact interface between mesosulfuron-methyl (yellow) and the ALS protein isoform II in S (WT) and R (Pro197-Ser + Trp574-Leu) Shepherd's-Purse plants (The protein contact surface is colored by H-bond donor or acceptor distribution; binding site amino acids are represented by sticks; intermolecular contacts are indicated by dashed lines, and the numbers are H-bond distance, Å. Amino acid numbering refers to Arabidopsis ALS protein).

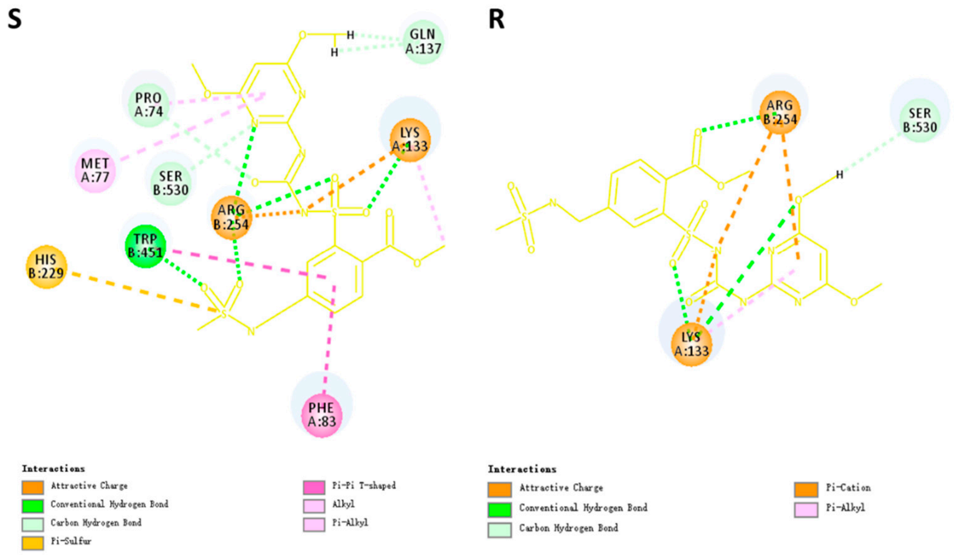

Figure S4. Two-dimensional diagram (2D-diagram) of interactions between mesosulfuron-methyl (yellow) and amino acid residues of the ALS protein isoform II in S (WT) and R (Pro197-Ser + Trp574-Leu) Shepherd's-Purse ALS protein (Amino acid residue Pro74, Met77, Phe83, Lys133, Gln137, His229, Arg254, Trp451 and Ser530 refers to Pro197, Met200, Phe206, Lys256, Gln260, His352, Arg377, Trp574 and Ser653 in Arabidopsis ALS protein, respectively).
